# Sequential Isolation of Microglia and Astrocytes from Young and Aged Adult Mouse Brains for Downstream Transcriptomic Analysis

**DOI:** 10.3390/mps5050077

**Published:** 2022-09-27

**Authors:** Ruchelle G. Buenaventura, Alex C. Harvey, Mark P. Burns, Bevan S. Main

**Affiliations:** Department of Neuroscience, Georgetown University Medical Center, Washington, DC 20057, USA

**Keywords:** sequential cell isolation, microglia, astrocytes, MACs, neuroinflammation, RNA

## Abstract

In aging, the brain is more vulnerable to injury and neurodegenerative disease, but the mechanisms responsible are largely unknown. Evidence now suggests that neuroinflammation, mediated by resident brain astrocyte and microglia populations, are key players in the generation of inflammatory responses and may influence both age related processes and the initiation/progression of neurodegeneration. Consequently, targeting these cell types individually and collectively may aid in the development of novel disease-modifying therapies. We have optimized and characterized a protocol for the effective sequential isolation of both microglia and astrocytes from the adult mouse brain in young and aged mice. We demonstrate a technique for the sequential isolation of these immune cells by using magnetic beads technology, optimized to increase yield and limit potential artifacts in downstream transcriptomic applications, including RNA-sequencing pipelines. This technique is versatile, cost-effective, and reliable for the study of responses within the same biological context, simultaneously being advantageous in reducing mice numbers required to assess cellular responses in normal and age-related pathological conditions.

## 1. Introduction

Microglia and astrocytes are key cellular components of the central nervous system (CNS), contributing to a variety of processes such as homeostasis, blood–brain barrier maintenance, synaptic remodeling and functional support of neurons [1,2]. Not only are these cells vital for proper CNS function, they are also resident immune cells that can initiate and sustain neuroinflammation, a key factor in the pathogenesis of many age-related neurodegenerative disorders. Activation of microglia and astrocytes in various injury or age-related paradigms can result in the secretion of cytokines, chemokines, and recruitment of circulating immune cells [3,4]. Temporally, these immune responses largely overlap and are crucial for repair, however if uncontrolled, these inflammatory responses exacerbate CNS injury and neurodegenerative pathology [5,6]. Despite knowledge of both the reparative and deleterious roles of these cells, heterogeneity occurs depending on disease paradigm, location, and spatial interaction between each other. Therefore, sequential isolation of microglia and astrocytes using fast isolation techniques that provide pure and sufficient cell specific yields without interference from other cell contaminants, is a powerful tool to study cellular immunophenotypes within the same brain tissue in models of neurodegeneration.

To achieve this in adult mouse brains, fluorescent-activated cell sorting (FACs) is a commonly used approach. FACs uses fluorescently labelled antibodies to identify subpopulations of cells in large numbers and at high purity [7,8]. However, it has its own inherent disadvantages. It requires a large starting number of cells in suspension, and as a result it may fail to isolate sufficient single cells from low quantity cell populations or small tissue samples [9]. The rapid flow of solution through the machine and non-specific fluorescent molecules also have the potential to damage and reduce the viability of sorted cells. Finally, FACs is comparatively time consuming and its reliance on expensive equipment and reagents results in high operational costs [10].

A related antibody-based technology, magnetic-activated cell sorting (MACs), is widely used as an alternative to FACs as it requires less time and less expensive equipment. It utilizes antibody-conjugated magnetic microbeads to select populations based on cell surface markers, resulting in a high specificity, high throughput, and cost-effective technique. These advantages have resulted in the refinement of MACs protocols for use on neonatal brain tissue [11,12,13], experiments requiring the isolation of a single cell type [14,15,16] and in-vitro studies [17,18,19]. However, detailed and reproducible MACs protocols to sequentially isolate multiple glial cell types (microglia followed by astrocytes), in the adult mouse brain, in both young and aged mice are limited.

Here we outline a protocol to sequentially isolate microglia and astrocyte populations from the adult mouse brain with high specificity using magnetic beads technology. This method is robust and reproducible, allowing for accurate analysis of microglial and astrocyte mediated neuroinflammatory mechanisms. Without the need of expensive equipment or fluorescently tagged cells, we show that RNA can be acquired at a low cost, while keeping high quality yield and purity for downstream transcriptomic applications. In addition, our protocol has been tested in young (3-months) and aged (18-months) mice, meaning it can be utilized in various fields of study including age-related models of neurodegeneration.

## 2. Experimental Design

**Figure 1:**
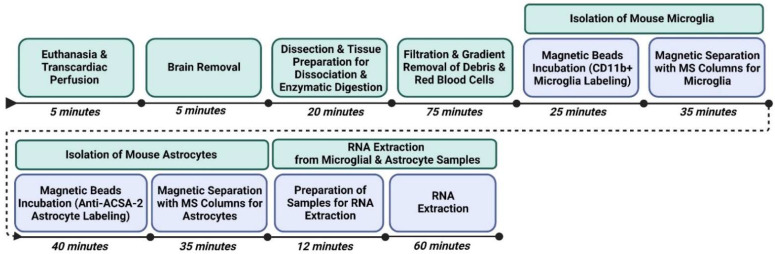
Sequential timeline of isolating microglia and astrocytes for downstream transcriptomic analysis with estimated time to completion. Note: RNA can be extracted at later time (within one week) once microglia and astrocyte samples are placed in TRIzol and can be stored at −20 °C.

All materials (Table 1), tools, equipment (Table 2), and solution recipes (Table 3) are listed as used in the protocol. In addition, the experimental design with an estimated timeline for sequentially isolating microglia and astrocytes for downstream transcriptomic analysis is presented in a schematic (Figure 1).

## 3. Procedure

### 3.1. Euthanasia and Transcardiac Perfusion

Note: Follow approved procedures of anesthesia and euthanasia in accordance with protocols approved with your relevant institution. The following is performed in accordance with protocols approved by the Georgetown University Animal Care and Use Committee.

**Figure 2:**
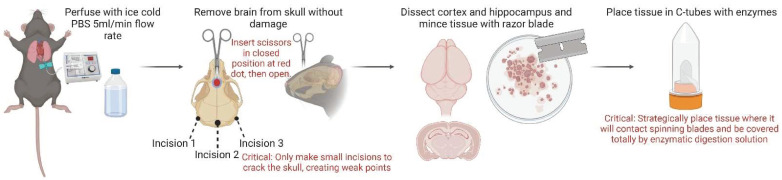
Schematic workflow of transcardiac perfusion, brain removal, dissection, and preparation of tissue for dissociation and enzymatic digestion. (Red denotes key steps that should be proceeded with carefully to ensure optimal performance, especially if dealing with small tissue quantity <50 mg).

1. Euthanize mouse (C57Bl/6J) via CO_2_ chamber at a flow rate of 4 L/min for approximately 3 min;
2. Place mouse on ice and to lift the upper ventral abdomen using Graefe forceps;
3. While holding Graefe forceps, make cutaneal incisions with light operating scissors, 3 cm horizontally and 3 cm vertically from the abdominal midline to expose the peritoneal cavity;
4. After cutaneal incisions have been made, confirm secondary euthanasia by lifting the xyphoid process with Graefe forceps and creating lateral incisions of the diaphragm along the upper thoracic line;
5. Make bilateral incisions of the thoracic vertebra and using Halsted mosquito forceps hold the xyphoid process above the chest cavity;
6. With the heart now exposed, insert a 21-gauge needle 5 mm into the left ventricle;
7. Turn on peristaltic pump to initiate transcardiac flush of ice-cold 1X PBS at 5 mL/min (see Table 3);
8. After turning on the pump, immediately incise the right atrium by 3 mm using micro dissecting scissors (see Figure 2);
9. Continue perfusion for approximately 2 min or until visible color change of liver indicating successful perfusion.

### 3.2. Brain Removal

1. Immediately post perfusion, decapitate the mouse with 6.5” operating scissors;
2. Cut the skin above the midline with micro dissecting scissors, to expose the skull and externally rotate the skin above the head;
3. Make bilateral incisions between the posterior midline and the parietal bone (approximately 10 mm) and between the posterior midline and the occipital bone (approximately 5 mm), creating a t-formation;
4. Using closed micro dissecting scissors, insert tip into the junction of the interfrontal suture and frontonasal sutures approximately 2 mm into the skull, then gently open the scissors to crack the calvaria midline down the interfrontal and sagittal suture, opening the skull into two hemispheres;
5. Using Graefe forceps, carefully separate both calvariae hemispheres away from brain;
6. Remove connective tissue from skull, and remove brain using curved forceps or micro spatula;
7. Place brain on a cold plate for immediate dissection of brain regions.

### 3.3. Dissection and Preparation of Tissue for Dissociation and Enzymatic Digestion

1. Position the brain on ice cold plate so that the cerebral cortices are facing upwards.

Using a sterile razor blade, hemisect the brain;

2. Using one brain hemisphere, turn over with cortex facing the cold block. Using small curved forceps, pinch out striatal/thalamus region leaving only cortical and hippocampal tissue;
3. Take a micro punch through cortical and hippocampal tissue (20–50 mg);
4. Using a sterile scalpel, slice/mince selected micro punch into small pieces;
5. Place segments of minced micro punch tissue around the cap of gentleMACS C Tube in a circular arrangement;
6. Prepare both enzyme mix 1 and 2 (see Table 3);
7. Add 1950 µL of enzyme mix 1 and 30 µL of enzyme mix 2 into gentleMACS C Tube;
8. Place cap with circular arranged tissue on gentleMACS C tube and close;
9. Tip C tube upside down, ensuring enzyme mix 1 and 2 solution is covering tissue;
10. Place closed C tube with tissue and enzymes on gentleMACS Octo Dissociator to proceed with gentleMACS Program 37C_ABDK_02;

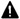
**CRITICAL STEP** Samples should be prepared directly after perfusion for optimal cell suspension performance. Keep samples on ice, especially when preparing larger sample sizes, to maintain optimal tissue integrity.

### 3.4. Filtration and Gradient Removal of Debris and Red Blood Cells (Figure 3)

1. Prepare MACS SmartStrainers (70 µm) by lightly prewetting surface with cold D-PBS;
2. After termination of the ABDK_02 program, detach C Tube from the gentleMACS Octo Dissociator with Heaters;
3. Resuspend sample by adding 10 mL of cold D-PBS to the gentleMACS C Tube. Close the tube and gently shake to collect samples at the bottom. Gentle inversions may be necessary to retrieve any remaining tissue on the lid;
4. Add components of C Tube through MACS SmartStrainers (70 µm) into a 50 mL conical tube;

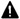
**CRITICAL STEP** Use the top end of a syringe plunger to mash and fully dissolve sample through strainer;
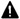
**CRITICAL STEP** Transfer samples from 50 mL conical tubes to new 15 mL conical tubes after filtration;

5. Centrifuge samples at 300× *g* for 10 min at 4 °C. Remove supernatant completely;
6. Resuspend cell pellet with 1550 µL of cold D-PBS in the 15 mL conical tube;
7. Add 450 µL of debris removal solution and mix well;
8. Gently overlay 2 mL of D-PBS into tube. Be careful not to mix phases;
  **OPTIONAL STEP** Slowly pipette D-PBS with the conical tube at a 45° angle to prevent phase mixing;

9. Centrifuge samples at 300× *g* for 10 min at 4 °C;
10. After three phases are formed, aspirate the top two phases gently and completely discard;

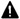
**CRITICAL STEP** Slowly remove at a 45° angle to increase accuracy;

11. With one phase remaining, fill tube with cold D-PBS to a final volume of 10 mL;
12. Gently invert three times;
13. Centrifuge at 1000× *g* for 10 min at 4 °C. Aspirate remaining supernatant completely;
14. Prepare 1X Red Blood Cell Removal Solution (see Table 3);
15. Resuspend pellet in 500 µL of cold 1X Red Blood Cell Removal Solution, and incubate for 10 min at 4 °C;
16. Add 5 mL of PB buffer and centrifuge at 300× *g* for 10 min at 4 °C. Remove remaining supernatant completely and proceed to microglial magnetic beads labelling.

**Figure 3:**
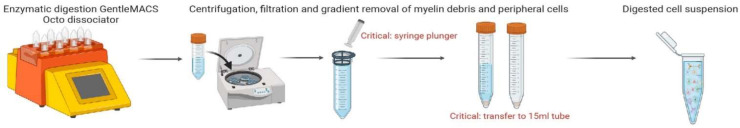
Schematic workflow of enzymatic digestion, centrifugation, and gradient removal of cellular debris. Critical modifications to protocol, highlighted in red, to allow for processing of small amounts of dissected brain tissue <50 mg.

### 3.5. Isolation of Mouse Microglia (Figure 4)

Magnetic Beads Incubation (CD11b^+^ microglia labeling):

1. Prepare PB buffer solution (see Table 3);
2. Resuspend pellet in 90 µL cold PB buffer by slowly pipetting up and down;
3. Add 10 µL CD11b (Microglia) MicroBeads, human and mouse mix well;
4. Incubate in the dark for 15 min at 4 °C;
5. Add 1 mL of cold PB buffer and mix well;
6. Centrifuge at 300× *g* for 5 min at 4 °C;
7. Remove supernatant and resuspend cells in 500 µL PB buffer;
  Magnetic Separation with MS Columns for Microglia

8. Place MS columns in the magnetic field of MACS Separator and prepare column by rinsing through 500 µL PB buffer. Discard flow through;

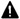
**CRITICAL STEP** Wait until column is completely empty before proceeding to next step;
**OPTIONAL STEP** To remove air bubbles from column after 500 µL rinse, use a needle syringe to pop bubbles;

9. Prepare 5 mL flow through collection tubes underneath MS columns for negative flow through collection;
10. Apply the 500 µL of CD11b^+^ labeled cells (from step 7 above) into MS column attached to magnetic separator;

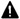
**CRITICAL STEP** To increase purity of microglia, apply negative flow through from each collection tube back into corresponding MS column for a second time;

11. Wash MS column with 500 µL PB buffer three times;
12. After wash is complete, remove 5 mL collection tubes (containing negative fraction);
13. Place 5 mL collection tubes (containing negative fraction) into a 15 mL falcon to allow for centrifugation; 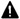 **CRITICAL STEP**
14. Centrifuge 15 mL falcon containing 5 mL collection tubes with negative flow though at 300× *g* for 10 min at 4 °C. Set aside for astrocyte labelling in Section 3.6;
15. Remove MS column containing CD11b^+^ bound cells from the separator, place it on corresponding collection tube, add 1 mL PB buffer, and immediately flush out to elute CD11b^+^ magnetically labeled cells with provided plunger;
16. Repeat step 14 with an additional 1 mL of PB buffer to increase yield;

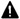
 **CRITICAL STEP**

17. Proceed to RNA extraction of eluted Cd11b^+^ microglia.

**Figure 4:**
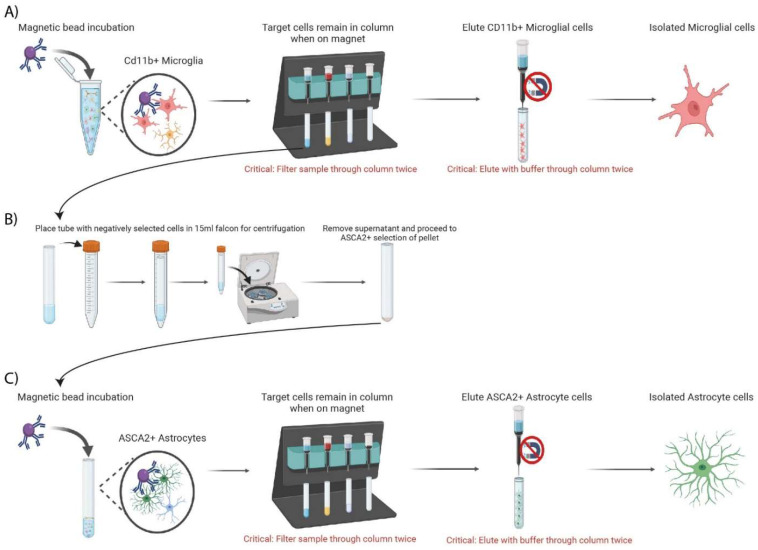
Schematic workflow of magnetic labeling for the sequential isolation of microglia and astrocytes. Following brain dissociation, debris, and red blood cell removal, (**A**) the purified homogenate is incubated with magnetically labelled anti-CD11b microbeads. Homogenate is then twice filtered through MS columns whilst attached to magnet. CD11b positively selected cells remain in the column and are then eluted into a fresh tube for microglial specific RNA extraction. The negative flow-through is collected for further processing. (**B**) Washed sample in negative flow-through columns are placed in 15 mL falcons to allow for centrifugation of cell pellet. Following centrifugation, supernatant is removed and pellet is processed for astrocyte isolation. (**C**) Sample incubated with anti-ACSA-2 microbeads, before astrocyte isolation and elution as described in (**A**).

### 3.6. Isolation of Mouse Astrocytes (Figure 4)

Sample preparation: Using forceps, carefully remove 5 mL reagent tubes from 15 mL conical tubes that were centrifuged in 3.5, step 13 and aspirate supernatant completely.

Magnetic beads incubation (Anti-ACSA-2 astrocyte labeling):

1. Resuspend pellet in 80 µL AstroMACS Separation Buffer (or PB buffer alternatively);
2. Add 10 µL of FcR Blocking Reagent, mix well, and incubate in the dark for 10 min at 4 °C;
3. Add 10 µL of Anti-ACSA-2 MicroBeads, mix well, and incubate in the dark for 10 min at 4 °C;
4. Add 1 mL of AstroMACS Separation buffer and place 5 mL reagent tubes back into 15 mL conical tubes and centrifuge at 300× *g* for 5 min at 4 °C;
5. Remove 5 mL reagent tubes, completely remove supernatant, and resuspend cells in 500 µL AstroMACS Separation Buffer;

Magnetic Separation with MS Columns for Astrocytes

6. Place new MS columns in the magnetic field of MACS Separator and prepare column by rinsing through 500 µL PB buffer. Discard flow through;

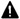
**CRITICAL STEP** Wait until column is completely empty before proceeding to next step;
**OPTIONAL STEP** To remove air bubbles from column after 500 µL rinse, use a needle syringe to pop bubbles;

7. Prepare 5 mL flow through collection tubes underneath MS columns for negative flow through collection;
8. Apply the 500 µL of ACSA2^+^ labeled cells (from step 5 above) into MS column attached to magnetic separator;

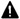
**CRITICAL STEP** To increase purity of astrocytes, apply negative selection back through MS column a second time;
**OPTIONAL STEP** To remove air bubbles from column entry disruption, use a needle syringe to remove obstructions;

9. Wash MS column with 500 µL PB buffer three times;
10. Remove MS column containing ACSA2^+^ bound cells from the separator, place it on corresponding collection tube, add 1 mL AstroMACS Separation buffer, and immediately flush out to elute ACSA2^+^ magnetically labeled cells with provided plunger.
11. Repeat step 11 with an additional 1 mL of AstroMACS separation buffer to increase yield; 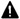 **CRITICAL STEP**
12. Proceed to RNA extraction of eluted ACSA2^+^ astrocytes.

### 3.7. RNA Extraction from Microglial and Astrocyte Samples (Figure 5, Figure 6 and Figure 7)

Preparation of samples for RNA Extraction

1. After magnetic separation of microglia or astrocytes, centrifuge samples at 1000× *g* for 5 min at 4 °C and remove supernatant (~2 mL);
2. Add 500 µL TRIzol, sonicate pellet completely, and transfer solution to 1.5 mL Eppendorf tubes. Proceed with subsequent RNA extraction. Samples can be stored at −20 °C for later extraction;

RNA Extraction

**Figure 5:**
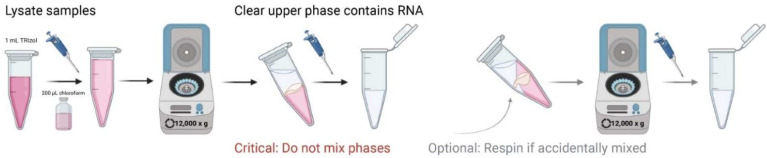
Schematic workflow of critical step in isolation of clear upper phase during phenol-chloroform (TRIzol) RNA extraction of isolated microglia and astrocytes.

3. Add 500 µL of TRIzol Reagent (total 1 mL) and incubate for 5 min at room temperature (RT);
4. Add 200 µL chloroform, thoroughly mix by shaking, and incubate at RT for 2 min.
5. Centrifuge samples at 4 °C for 15 min at 12,000× *g*;
6. The mixture separates into 3 layers which include a colorless upper phase, white interphase, and pink phenol-chloroform lower phase;

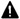
**CRITICAL STEP** In a 45° angle, being careful not to mix phases together, remove clear top layer containing RNA into a new tube. For optimal purity, closely view tube for phase movement and do not transfer contaminated mixed-phase solution (Figure 5);
**OPTIONAL STEP** Should any of the three layers be accidentally mixed, centrifuge samples again at 4 °C for 15 min at 12,000× *g* and repeat the upper-phase extraction, which contains RNA (Figure 5);

7. Add 500 µL of 100% isopropanol to each sample, invert tubes three times, and incubate in 4 °C for 10 min;
8. Centrifuge in 4 °C for 10 min. Tubes should be spun with the hinged cap side up, which will be used as a physical guideline for identifying pellets at later stages. It is important to maintain this tube angle in all centrifugation steps;
9. Due to use of a 45° rotor, a small, white translucent, gel-like pellet should appear after centrifugation along the bottom of the tube on the same side of the cap’s hinge. Carefully discard supernatant while maintaining pellet in the tube;

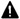
**CRITICAL STEP** It is recommended to grade down pipette sizes for increased precision of supernatant removal;
**OPTIONAL STEP** For smaller samples, it is recommended to perform procedure alongside a larger tissue sample (50–100 mg) as a reference control for pellet location;

**Figure 6:**
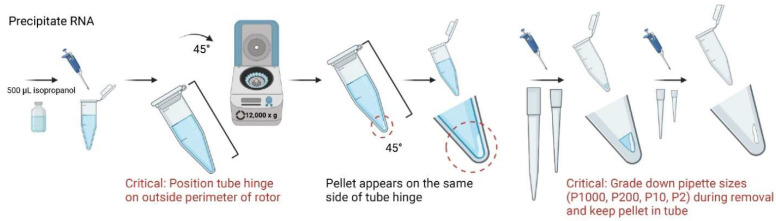
Schematic workflow of critical step in removal of supernatant and keeping clear RNA pellet in tube during RNA extraction of isolated microglia and astrocytes.

10. Resuspend pellet with 1 mL of 75% ethanol, vortex, and centrifuge in 4 °C for 5 min at 7500× *g*;
11. A thin gel-like pellet should appear after centrifugation in the same 45° location. Discard as much supernatant as possible while maintaining visible sight of pellet. If pellet is not clearly visible, use a larger tissue sample for reference of location;

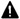
**CRITICAL STEP** For optimal purity, leave a small amount of supernatant (~ 20 µL) in the tube and centrifuge at 7500× *g* in 4 °C for 5 min again. After spin, discard supernatant with P2 or P10 pipette;
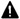
**CRITICAL STEP** It is recommended to grade down pipette sizes for increased precision of supernatant removal and preservation of RNA;

12. Add 15 µL of nuclease-free H_2_O to each sample and vortex. Place samples in −80 °C for storage until use;

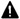
**CRITICAL STEP** To prevent RNA degradation and evaporation, do not leave tube cap open for an extended period of time;

13. Add 1 µL of extracted RNA to NanoDrop One/OneC Microvolume UV-Vis Spectrophotometer to determine concentration. Store at -80C until further use.

**Figure 7:**
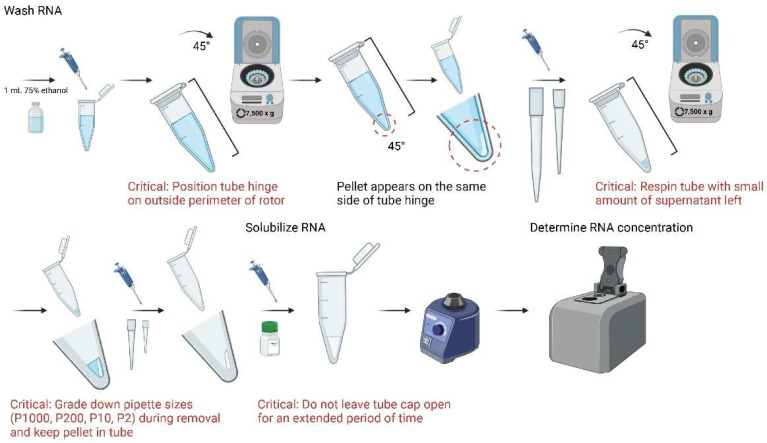
Schematic workflow of final key steps in the isolation of RNA from microglia and astrocytes.

## 4. Expected Results and Discussion

In many experimental paradigms, the ability to isolate multiple CNS cell types from a single homogenate is advantageous. It allows for exploration of cellular morphology with the relatively gently nature of MACs isolations preserving cell integrity and networks of processes [20,21]. Isolated microglia exhibit a typical longitudinal bipolar cell body with ramified pseudopodia, while astrocytes display branched cellular processes and arborizations that allow for extra somatic proteins to be visualized [13,17,22]. Further adding to the advantageous nature of this technology, is its high throughput capacity in a time and cost-effective manner. Seminal results show isolated microglia and astrocytes display high purity (94% and 96%, respectively), with a total number of 4 × 10^5^ astrocytes and 4.2 × 10^5^ microglia obtained from one adult mouse brain each [23]. Optimization of protocols have seen these numbers refined, with microglial cell counts of 50,000–200,000 from bilateral hippocampus [24] and single hemispheres [25]. Viability of microglia and astrocytes separated by MACS are routinely observed to be greater than 90% [17,23]. Of relevance to this study, MACs isolations can also be used to perform multi-omic analyses of subpopulations of immune cells, enhancing our understanding of complex cellular networks.

Since RNA sequencing was first introduced in 2008 [26,27], it has become a common tool in molecular biology, influencing almost every aspect of our understanding of genomic function. Although outside the methodological scope of this manuscript, the standard workflow consists of RNA extraction followed by mRNA enrichment or ribosomal RNA depletion. Subsequently, cDNA synthesis and creation of an adaptor-ligated sequencing library is performed before libraries are sequenced on a high-throughput machine platform to a read depth of 10–30 million reads per sample. The final steps are bioinformatic based, aligning sequencing reads to the transcriptome of the species of interest, before normalizing samples and investigating levels of differential gene expression (reviewed in [28]). Indeed, to date, almost 100 distinct methods have been derived from the standard RNA-seq protocol [29]. This combined with the affordability and accessibility of sequencing platforms provided by commercially available pharmaceutical/biotech companies, as well as academic shared-resources, means that RNA sequencing has now emerged as a commonplace technique in experimental designs across research fields.

Here, we describe an application of immuno-magnetic cell separation that allows for the sequential isolation of glial cell populations (astrocytes and microglia) from the adult mouse brain for downstream transcriptomic analysis. This technique is cost-effective and modifiable for the study of multiple responses within the biological context of a single mouse brain microenvironment. It is also advantageous in terms of reducing the number of mice required to assess cellular responses in models with limited tissue for analysis (e.g., ipsilateral brain injury).

**Figure 8:**
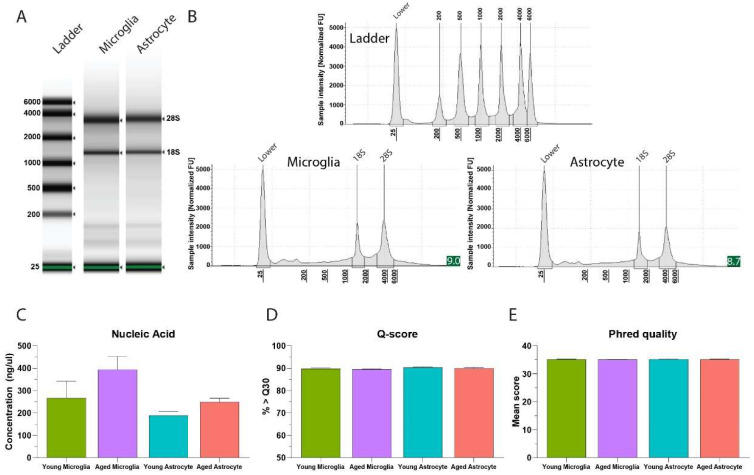
RNA quality, integrity, and yield for transcriptomic applications after sequential cell specific isolation. (**A**) Representative electrophoresis demonstrating RNA quality of sequentially extracted microglia and astrocyte samples from combined cortical and hippocampal tissue from the adult mouse brain. (**B**) Electropherogram showing 18S and 28S peaks of microglia and astrocyte samples, with integrity scores of 9.0 and 8.7 respectively evidencing high quality RNA with minimal degradation. (**C**) RNA yields following sequential isolation of microglia and astrocytes from young and aged brains. Quality control of transcriptomic sequencing of isolated microglial and astrocytes demonstrated by (**D**) Q-score > 30 and (**E**) mean Phred scores.

Using this sequential isolation protocol, we demonstrate the ability to isolate sufficient quantities of RNA suitable for downstream transcriptomic applications. Chip based capillary electrophoresis of RNA from isolated microglia and astrocytes shows high quality and minimal degradation as evidenced by high ratios of 18S to 28S ribosomal bands, and minimal baseline signal between peaks (Figure 8B). Electropherogram visualization and integrity algorithms routinely classified samples as having integrity scores between 8–9, and total yields displaying concentrations of 200 ng/µL or higher (Figure 8C). Of importance, low-quality RNA, can substantially affect the sequencing results (e.g., uneven gene coverage, 3′–5′ transcript bias) and lead to erroneous biological conclusions [30]. We show that this protocol not only results in high quality RNA, but downstream transcriptomic control measures show high %Q- and Phred scores (Figure 8D,E), evidencing high quality sequencing data (99.9% base call accuracy), with low rates of false positive gene expression.

**Figure 9:**
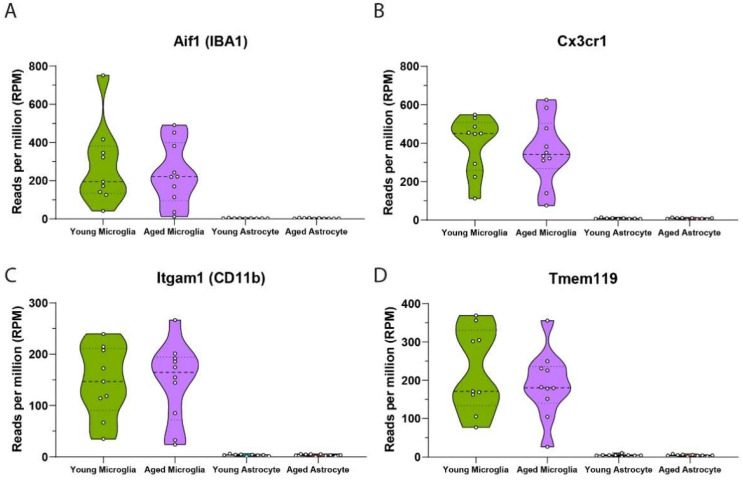
Transcriptomic validation of microglia isolated from adult mouse brain. mRNA analysis shows isolated microglial populations have high expression of microglial specific markers (**A**) Aif1, (**B**) Cx3cr1, (**C**) Itgam1 and (**D**) Tmem119 (young microglia n = 9, young astrocyte n = 9, aged microglia n = 10, aged astrocyte n = 10). Data from 3 independent experiments run on different days, a total of n = 3/day for young mice and n = 3, 3, 4 per day for aged mice.

Advancements in sequencing-based technologies and comparative transcriptomic studies have generated useful resources for identifying the molecular signatures of many CNS cell types, including microglia and astrocytes [31,32,33]. As a result, we can use transcriptomic profiling to determine the composition of cell populations. In both young and aged isolated microglia, we confirm the elevated expression of microglial specific markers, indicating highly enriched microglial populations (Figure 9A–D).

To achieve this result, the order of sequential isolation is of critical importance. Given microglia constitute only 5–10% of total brain cells [5], Cd11b^+^ magnetic labelling and selection should occur first to provide the highest probably of cell capture at sufficient yields. In addition, we pass the Cd11b^+^ cell homogenate through the MS filter columns twice before the elution of positively selected cells. We believe this step is critical to increase the purity and yield of Cd11b^+^ microglial cells selected, especially given our preparations are from a relatively small starting amount of tissue (singular micro punch <50 mg, as would be used in traumatic brain injury models of ipsilateral injury [34]).

**Figure 10:**
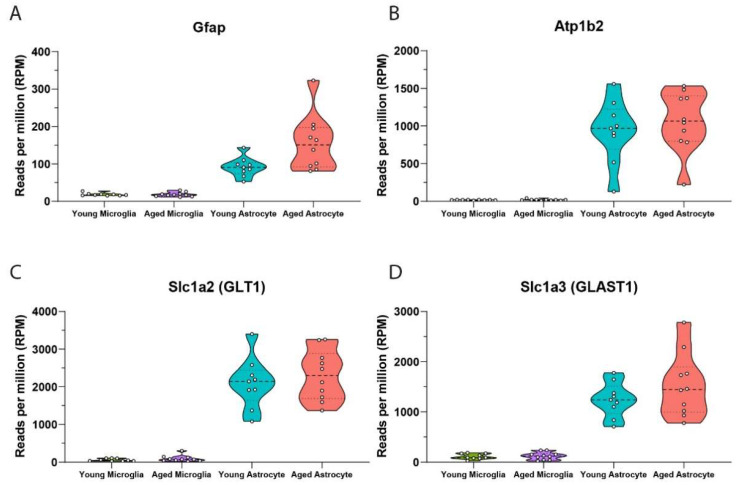
Transcriptomic validation of sequentially isolated astrocytes from adult mouse brain. mRNA analysis shows astrocytes that are isolated after microglial preparations have high expression of astrocyte specific markers (**A**) Gfap, (**B**) Atp1b2, (**C**) Slc1a2 and (**D**) Slc1a3 (young microglia n = 9, young astrocyte n = 9, aged microglia n = 10, aged astrocyte n = 10). Data from 3 independent experiments run on different days, a total of n = 3/day for young mice and n = 3, 3, 4 per day for aged mice.

Following Cd11b^+^ microglial selection, the negative flow-through is collected and used for subsequent isolation of astrocytes. This negative collection is gently centrifuged before astrocytes are isolated using ASCA2^+^ microbeads selection. Similar to prior microglial preparations, incubated ASCA2^+^ volumes are passed through columns twice to increase yield through positive selection magnetic binding. We confirmed this sequential step of the protocol is specific for astrocyte populations, evidenced by the expression of elevated astrocyte markers Gfap, Atp1b2, Slc1a2 and Slc1a3 in both young and aged astrocytes, but not in microglial preparations (Figure 10A–D). In addition to confirming positive selection of astrocytes using this method, we also show that isolated young and aged astrocytes display elevated levels of Atp1b2, in agreement with a previous study identifying ATP1B2 as the target epitope of ASCA2 antibody binding [14].

## 5. Conclusions

Our modified magnetic cell sorting technique allows for sequential isolation of microglia and astrocytes that is effective in both the young and aged adult mouse brain. This protocol allows for the cost-effective assessment of cellular interactions in the biological context of the individual mouse brain, providing a powerful tool for the holistic analysis of CNS responses. We show that in our experience, a single mouse cortex and/or hippocampus is sufficient to isolate cells for downstream transcriptomic analysis, allowing for the ethical reduction and refinement of the experimental animals required.

## Figures and Tables

**Table 1:** Detailed information on required materials.

Name	Source	Identifier	Location
**Stock Solutions and Kits** Phosphate Buffered Saline 10X Solution Adult Brain Dissociation Kit, mouse and rat			
	Fisher Scientific	BP399-20	Fair Lawn, NJ, USA
	Miltenyi Biotec B.V. & Co. KG	130-107-677	Bergisch Gladbach, DE
Enzyme P (2.5 mL) Buffer Z (50 mL) Buffer Y (1.5 mL) Enzyme A (1 vial lyophilized powder) Buffer A (1 mL) Red Blood Cell Removal Solution 10X (5 mL) Debris Removal Solution (45 mL)			
		
CD11b (Microglia) MicroBeads, mouse/human (1 mL) Anti-ACSA-2 MicroBead Kit mouse (2 × 1 mL) FcR blocking reagent (1 mL) Anti-ACSA-2 MicroBeads (1 mL)	Miltenyi Biotec B.V. & Co. KG	130-093-634	Bergisch Gladbach, DE
	Miltenyi Biotec B.V. & Co. KG	130-097-678	Bergisch Gladbach, DE
		
		
AstroMACS Separation Buffer (100 mL) Dulbecco’s Phosphate Buffered Saline (with calcium) MACS BSA Stock Solution TRIzol Reagent Chloroform Isopropanol Ethanol, Anhydrous Nuclease-Free Water (DEPC Treated)	Miltenyi Biotec B.V. & Co. KG Thermo Fischer Scientific	130-117-336 14040117	Bergisch Gladbach, DE Waltham, MA, USA
	Miltenyi Biotec B.V. & Co. KG Invitrogen Fisher Scientific Fisher Scientific Fisher Scientific Invitrogen	130-117-336 15596026 C298-500 A416-4 A405P-4 AM9906	Bergisch Gladbach, DE Waltham, MA, USA Fair Lawn, NJ, USA Fair Lawn, NJ, USA Fair Lawn, NJ, USA Waltham, MA, USA

**Table 2:** Detailed information on tools and equipment.

Name	Source	Identifier	Location
**Tools** 21 G x 1” needle Graefe forceps Light operating scissors Halsted mosquito forceps Operating scissors 6.5” Micro dissecting scissors 4.5” Scalpel handle Scalpel blades **Equipment** Fisherbrand Variable-Flow Peristaltic Pump Dissection cold plate OctoMACS Separator attached to MultiStand MS Columns 5 mL Tubes for MS Columns gentleMACS C Tubes gentleMACS Octo Dissociator with Heaters MACS SmartStrainers 70 µm 50 mL Conical Screw Cap Tubes, Grenier Bio-One 15 mL Conical Screw Cap Tubes, Grenier Bio-One 5 mL Falcon Round Bottom Polystyrene Test Tube Centrifuge (refrigerated) Vortex Mixer Sonicator 1.5 mL Microcentrifuge Tubes (clear) NanoDrop One Microvolume Spectrophotometer	Covetrus Roboz Surgical Store Roboz Surgical Store Roboz Surgical Store Roboz Surgical Store Roboz Surgical Store Roboz Surgical Store Roboz Surgical Store Fisher Scientific Cell Path Miltenyi Biotec B.V. & Co. KG Miltenyi Biotec B.V. & Co. KG Miltenyi Biotec B.V. & Co. KG Miltenyi Biotec B.V. & Co. KG Miltenyi Biotec B.V. & Co. KG Miltenyi Biotec B.V. & Co. KG USA Scientific, Inc USA Scientific, Inc Corning Eppendorf, model 5810R Labnet International, Inc. QSonica, LLC USA Scientific, Inc Thermo Fisher Scientific	060760 RS-5138 RS-6752 RS-7111L RS-6824 RS-5912 RS-9843 RS-9801-11 13-876-2 JRI-0100-00A 130-042-109 130-042-201 130-091-598 130-093-237 130-095-427 130-110-916 5622-7270 5618-8261 352054 022627040 S0100-VX100 Q55-100 1615-5510 ND-ONE-W	Dublin, OH, USA Gaithersburg, MD, USA Gaithersburg, MD, USA Gaithersburg, MD, USA Gaithersburg, MD, USA Gaithersburg, MD, USA Gaithersburg, MD, USA Gaithersburg, MD, USA Fair Lawn, NJ, USA Newtown, UK Bergisch Gladbach, DE Bergisch Gladbach, DE Bergisch Gladbach, DE Bergisch Gladbach, DE Bergisch Gladbach, DE Bergisch Gladbach, DE Ocala, FL, USA Ocala, FL, USA Glendale, AZ, USA Enfield, CT, USA Woodridge, NJ, USA Newtown, CT, USA Ocala, FL, USA Waltham, MA, USA

**Table 3:** Detailed information for solutions (to be prepared immediately before use).

Name	Recipe
1 × PBS Enzyme Mix 1 Enzyme Mix 2 D-PBS PB Buffer 1x Red Blood Cell Removal Solution	1:10 dilution of Phosphate Buffered Saline 10X Solution with dH_2_O 50 µL Enzyme P and 1900 µL Buffer Z (prepare fresh) 20 µL Buffer Y and 10 µL Enzyme A (prepare fresh) 1:10 dilution of Dulbecco’s Phosphate Buffered Saline (with calcium) with dH_2_O and cool to 4 °C 1:20 dilution of MACS BSA Stock Solution with D-PBS and cool to 4 °C (or prepare a solution with pH of 7.2 with phosphate-buffered saline (PBS), 0.5% bovine serum albumin (BSA), and 2 mM EDTA) 1:10 dilution of Red Blood Cell Removal Solution (10X) with cold dH_2_O (prepare fresh)

## Data Availability

Data supporting reported results is available by emailing the corresponding author.
